# iNOS-Dependent Increase in Colonic Mucus Thickness in DSS-Colitic Rats

**DOI:** 10.1371/journal.pone.0071843

**Published:** 2013-08-19

**Authors:** Olof Schreiber, Joel Petersson, Tomas Waldén, David Ahl, Stellan Sandler, Mia Phillipson, Lena Holm

**Affiliations:** Department of Medical Cell Biology, Uppsala University, Uppsala, Sweden; Charité-University Medicine Berlin, Germany

## Abstract

**Aim:**

To investigate colonic mucus thickness *in vivo* in health and during experimental inflammatory bowel disease.

**Methods:**

Colitis was induced with 5% DSS in drinking water for 8 days prior to experiment, when the descending colonic mucosa of anesthetized rats was studied using intravital microscopy. Mucus thickness was measured with micropipettes attached to a micromanipulator. To assess the contributions of NOS and prostaglandins in the regulation of colonic mucus thickness, the non-selective NOS-inhibitor L-NNA (10 mg/kg bolus followed by 3 mg/kg/h), the selective iNOS-inhibitor L-NIL (10 mg/kg bolus followed by 3 mg/kg/h) and the non-selective COX-inhibitor diclofenac (5 mg/kg) were administered intravenously prior to experiment. To further investigate the role of iNOS in the regulation of colonic mucus thickness, iNOS −/− mice were used.

**Results:**

Colitic rats had a thicker firmly adherent mucus layer following 8 days of DSS treatment than untreated rats (88±2 µm vs 76±1 µm). During induction of colitis, the thickness of the colonic mucus layer initially decreased but was from day 3 significantly thicker than in untreated rats. Diclofenac reduced the mucus thickness similarly in colitic and untreated rats (−16±5 µm vs −14±2 µm). While L-NNA had no effect on colonic mucus thickness in DSS or untreated controls (+3±2 µm vs +3±1 µm), L-NIL reduced the mucus thickness significantly more in colitic rats than in controls (−33±4 µm vs −10±3 µm). The importance of iNOS in regulating the colonic mucus thickness was confirmed in iNOS−/− mice, which had thinner colonic mucus than wild-type mice (35±3 µm vs 50±2 µm, respectively). Furthermore, immunohistochemistry revealed increased levels of iNOS in the colonic surface epithelium following DSS treatment.

**Conclusion:**

Both prostaglandins and nitric oxide regulate basal colonic mucus thickness. During onset of colitis, the thickness of the mucus layer is initially reduced followed by an iNOS mediated increase.

## Introduction

A continuous mucus layer covers the epithelium of the gastrointestinal tract extending from the stomach to the colon. The colonic mucus is composed of gel-forming Muc2 mucins secreted by mucus producing goblet cells scattered throughout the colonic epithelium [1], [2], [3]. This mucus comprises an important barrier that prevents bacteria and other inflammatory agents from invading the mucosa, which is demonstrated by genetically modified mice. These mice are completely (Muc2-/−) [4], [5] or partially deficient in gel-forming Muc2 protein (C3GnT [18]) and spontaneously develop colitis and later colorectal tumors, in addition to being more susceptible to DSS-induced colitis. We have shown that the colonic mucus can be divided into two layers, an outer layer which is easily removed by suction, the loosely adherent mucus, and a firmly adherent layer that cannot be removed unless harming the underlying mucosa [2], [6]. The thickness of the mucus layer is the result of mucus secretion and erosion by mechanical shear and bacterial enzymatic degradation [7]. Little is known about the regulation of colonic mucus thickness mainly due to technical difficulties and the lack of intestinal in vitro culture systems that replicate the complexity of the in vivo mucus barrier.

Inflammatory bowel disease, IBD, is comprised of a group of chronic autoimmune inflammatory conditions with unknown etiology, e.g. ulcerative colitis and Crohn’s disease. The experimental model used in this study resembles ulcerative colitis in terms of localisation and clinical symptoms, since only the mucosa of the colon becomes affected [8]. The colon houses a vast number of bacteria, the commensal flora, which has been implicated in the pathogenesis of IBD [9]. Under normal circumstances, these bacteria do not cause clinical inflammation, at least partly due to the firmly adherent mucus layer. In contrast to the firmly adherent mucus layer, which has been shown to exclude a majority of the colonic bacteria [2], [10], the loosely adherent mucus harbours much of the commensal microbiota and prevent it from being lost with the faeces. Further studies have demonstrated that the bacterial products PGN and LPS stimulate mucus secretion [3]. Thus bacteria induce its own environment for colonization, but also the protective barrier to avoid contact with the epithelium.

Both the nitric oxide (NO) system and prostaglandins have been shown to regulate many events in the gastrointestinal tract. We have earlier shown that the endothelial NO-synthase, eNOS is involved in the increased colonic mucosal blood flow observed in colitic rats [11]. In addition, prostaglandins have been shown to stimulate mucin secretion thereby enhancing colonic mucosal barrier function [12], [13]. We have also shown that gastric mucus secretion, containing Muc 5AC and Muc 6 mucins, is influenced by both prostaglandins and NO, and these mediators have been shown to play different roles in secretion of the different mucus layers [14]. Little is known about the Muc 2 colonic mucus thickness during the onset of colitis, partially depending on the heterogeneous nature of inflammation signifying the importance of concomitant studies of the mucus and mucosal inflammation performed at the same site. Human studies in ulcerative colitis patients have shown an altered and less effective mucus barrier [15], [16], [17], [18]. Other studies in mice indicate that mucus thickness is reduced when disease activity is increased [3].

In the current study, the influence of onset of DSS-induced colitis on colonic mucus was investigated in vivo in rats. Further, the involvement of prostaglandins and nitric oxide were studied in the regulation of mucus thickness in the basal situation as well as during colitis.

## Materials and Methods

### Ethics Statement

All animal experiments were approved by the Swedish Laboratory Animal Ethical Committee in Uppsala (animal experiments numbers C286/8 and C110/8) and were conducted in accordance with guidelines of the Swedish National Board for Laboratory Animals.

### Animals

Eighty-five male Sprague-Dawley rats (B&K, Sollentuna, Sweden) weighing between 190 and 290 g (weight before treatment), 18 male C57Bl/6 mice and 9 male C57Bl/6x129SvEv mice deficient in iNOS, were kept under standardized conditions at a temperature of 21–22°C and with 12 h light and 12 h dark a day. The animals were allowed to acclimatize for 1 week before the experiments were commenced.

### Rat Preparation

The rats were anesthetized with 120 mg kg^−1^ body weight of 5-ethyl-5-(1-methylpropyl)-2-thiobutabarbital sodium (Inactin®, Sigma, St. Louis, MO), given intraperitoneally. The body temperature was maintained at 37–38°C using a heating pad controlled by a rectal thermistor probe. To facilitate spontaneous breathing a short PE-200 cannula was placed in the trachea. To monitor blood pressure, a PE-50 cannula containing heparin (Leo Pharma AB, Sweden, 12.5 IU ml^−1^ dissolved in 0.9% saline) was placed in the right femoral artery, and the right femoral vein was catheterized to enable a continuous infusion of Ringer’s solution (120 mM NaCl, 25 mM NaHCO_3_, 2.5 mM KCl and 0.75 mM CaCl_2_) at a rate of 1 ml per hour during the experiment. In some experiments the right femoral vein was also the route for administration of the unselective NOS-inhibitor N^ω^-nitro-L-arginine (L-NNA) (Sigma, St Louis, MO), the iNOS inhibitor L-N^6^-(1-iminoethyl)-lysine (L-NIL) (Sigma, St. Louis, MO) or the COX inhibitor diclofenac, (Voltaren, Novartis, Täby, Sweden) in the Ringeŕs solution. The colon was exteriorized through a midline abdominal incision and 1–2 cm of the descending colon was opened longitudinally. The rat was placed on its right side on a Lucite microscope stage. The colon was everted and loosely draped over a truncated cone. A double-bottom mucosal chamber with warm circulating water and a hole in the bottom was fitted over the mucosa, exposing approximately 0.5 cm^2^ of the mucosal surface through the hole. The junction was sealed with silicon grease (Dow Corning high vacuum grease, Dow Corning GmbH, Weisbaden, Germany). The mucosal chamber was filled with approximately 5 ml of saline at 37°C to keep the tissue moist and warm. This model is further described by Atuma et al. [6]. At the end of the experiments, the anesthetized rats were euthanized by an intravenous injection of 2 ml saturated potassium chloride.

### Mouse Preparation

The mice were anesthetized with spontaneous inhalation of isoflurane (Forene, Abbott Scandinavia, Solna, Sweden). The inhalation gas (≈2.2% isoflurane) was continuously administered through a breathing mask (Simtec Engineering, Askim, Sweden) in a mixture of 40% oxygen and 60% nitrogen. The body temperature was maintained at 37–38°C using a heating pad controlled by a rectal thermistor probe. The descending colon was exteriorized in the same way as the rat colon, but exposing approximately 0.05 cm^2^ of the mucosal surface through the hole. In 4 of the C57Bl/6 and 4 of the iNOS−/− mice diclofenac (Voltaren, Novartis, Täby, Sweden) was injected through a PE-50 cannula placed in the right jugular vein. At the end of the experiments, the anesthetized mice were euthanized by cervical dislocation.

### Induction of Colitis

Rats were given 5% (wt/wt) Dextran Sulphate Sodium (DSS 37–40 kilodaltons; TdB Consultancy, Uppsala, Sweden) in the drinking water for 8 days. The control rats for the DSS group were untreated.

### Assessment of Severity of Colitis

The severity of the colitis was assessed on the basis of clinical parameters such as stool consistency and fecal blood content. This severity, together with any weight loss was scored daily with the use of Disease Activity Index (DAI), a scoring method described in detail by Cooper et al [19].

### Rat Experimental Protocol

After the surgical preparation the rats were given 1 hour to stabilize before the mucus measurements began. The rats were divided into control groups and DSS colitis groups, and these two groups were further divided into subgroups in respect to treatment: I) Saline, II) the NOS inhibitor L-NNA (10 mg kg^−1^ bolus in 1.0 ml followed by an i.v. infusion of 3 mg kg^−1^h^−1^ infused at 1.0 ml h^−1^), III) the iNOS inhibitor L-NIL (10 mg kg^−1^ bolus in 1.0 ml followed by an i.v. infusion of 3 mg kg^−1^h^−1^ infused at 1.0 ml h^−1^) and IV) the cyclooxygenase inhibitor diclofenac (5 mg kg^−1^ bolus in 1.0 ml). To investigate colonic mucus thickness during colitis induction, we also measured mucus thickness in 28 rats, 4 per day, on days 1–7 of the DSS-regimen.

### Mouse Experimental Protocol

After the preparation the mice were given 1 hour to stabilize before the mucus measurements began. Firmly adherent mucus thickness was measured in 18 control C57Bl/6 mice and 9 iNOS−/− C57Bl/6x129SvEv mice. In 4 of the control and 4 of the iNOS−/− mice, diclofenac (5 mg/kg in 1.0 ml) was given as a bolus injection i.v. in the jugular vein and the mucus thickness was measured 60 minutes later.

### Mucus Measurements

Glass micropipettes (custom glass tubing; OD, 1.2 mm; ID, 0.6 mm; Rederick Haer, Brunswick, ME) were pulled to a tip diameter of 1–2 µm. The micropipettes were siliconized to obtain a non-adhesive surface, which facilitates repeated measurements. After the animals had stabilized for one hour, total mucus thickness was measured. Then the loosely adherent mucus was removed by suction and the firmly adherent mucus thickness was measured. By subtracting the firmly adherent mucus thickness from total mucus thickness, the loosely adherent mucus thickness was obtained. The animal were administered either saline, L-NNA, L-NIL or diclofenac, and a second mucus thickness measurement was performed after one hour. Before each measurement, the mucus gel was covered with enough carbon particles (activated charcoal, extra pure, Kebo Lab, Huddinge, Sweden) suspended in saline to visualize the surface of the otherwise near-transparent gel. The micropipettes were held by a micromanipulator (Leitz, Wetzlar, Germany) and pushed into the mucus gel at an angle (*a*) of 30–35° to the cell surface. The distance (*l*) from the luminal surface of the mucus layer to the epithelial cell surface of the mucosa was measured with a “digimatic indicator” (IDC series 543; Mitutoyo, Tokyo, Japan) connected to the micromanipulator. The mucus gel thickness (*T*) could then be calculated from the formula *T* = *l* × sin *a*. The measurements were made at five different spots over the colonic mucosal surface and a mean value was calculated. The same spots were used for the second measurements after intervention. For detailed description of the method for mucus measurements see [20].

### Histology

Samples of the distal colon from controls and DSS-colitic rats were fixed in 4% formalin, processed, embedded in wax, sectioned and stained with Periodic Acid Shiff’s stain. The number of PAS-positive cells, as well as area per goblet cell was evaluated.

### Immunohistochemistry

Distal colon tissue samples in Neg50 OCT-medium (Cellab, Stockholm, Sweden), from 4 control- and 4 DSS-rats, were snap-frozen in liquid nitrogen, sectioned (8 µm), fixated in ice-cold methanol, permeabilized (0.05% Triton X) before antibody incubation. Thereafter, the slides were incubated with a primary antibody targeting iNOS (ab31630, Abcam, Cambridge, UK) and thereafter with secondary antibody (Alexa 488, Life Technologies). Nuclei were stained with Hoechst 33345 (Life Technologies). Images were acquired using a laser scanner confocal microscopy (Nikon C1 on a TE-2000-U base with Plan APO VC 20x/0.75) and analyzed using EZ-C1 software (Nikon) and ImageJ (NIH, Bethesda, MD).

### Statistical Analysis

All values are expressed as means ± SEM. Multiple comparisons were performed using one way ANOVA in R (Version 2.11.1; R Foundation for Statistical Computing, Vienna, Austria). If the ANOVA was significant, Fisher’s PLSD *post hoc* test was performed. Single comparisons were performed using Student’s t-test. For all comparisons, P<0.05 was considered statistically significant.

## Results

### The Firmly Adherent Mucus Thickness Increased during Established Colitis in Rats

**Figure 1:**
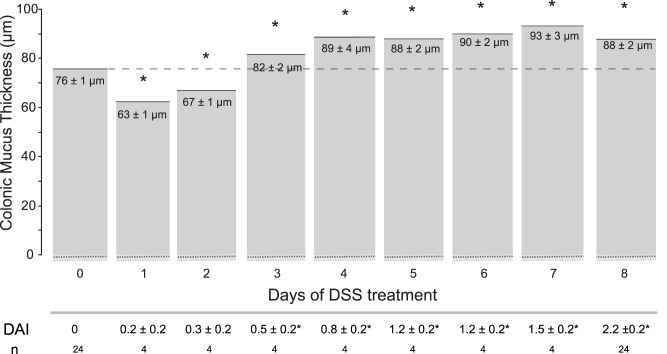
Firmly adherent mucus thickness in DSS-treated rats. Firmly adherent mucus thickness during 8 days following colitis induction with DSS. The initial decrease in mucus thickness was followed by an increased mucus thickness from day 3 compared to controls, day 0 (p<0.05). Disease Activity Index was also significantly increased from day 3 and onward.

During established colitis at 8 days following induction of DSS treatment, the firmly adherent mucus thickness was significantly thicker (88±2 µm, n = 24) compared to in untreated rats (76±1 µm, n = 24, Fig. 1, day 0), while no difference in the thickness of the loosely adherent mucus was observed (211±39 µm vs 183±33). Interestingly, when the mucus layer was investigated during onset of colitis by measuring its thickness in different individuals treated with DSS for 1 up to 8 days, we found that the mucus thickness initially decreased (63±1 µm and 67±1 µm at day 1 and 2, respectively) compared to basal levels in untreated rats (76±1 µm). This initial mucus reduction was followed by a gradual increase, resulting in a significantly thicker mucus layer from day 3 compared to basal levels (Fig. 1). The increased mucus thickness coincided with an elevated disease activity index (DAI) from day 3 in response to DSS treatment. Untreated rats had a DAI of 0 during the period of 8 days.

### The Number of Goblet Cells Increased in DSS Colitis

**Figure 2:**
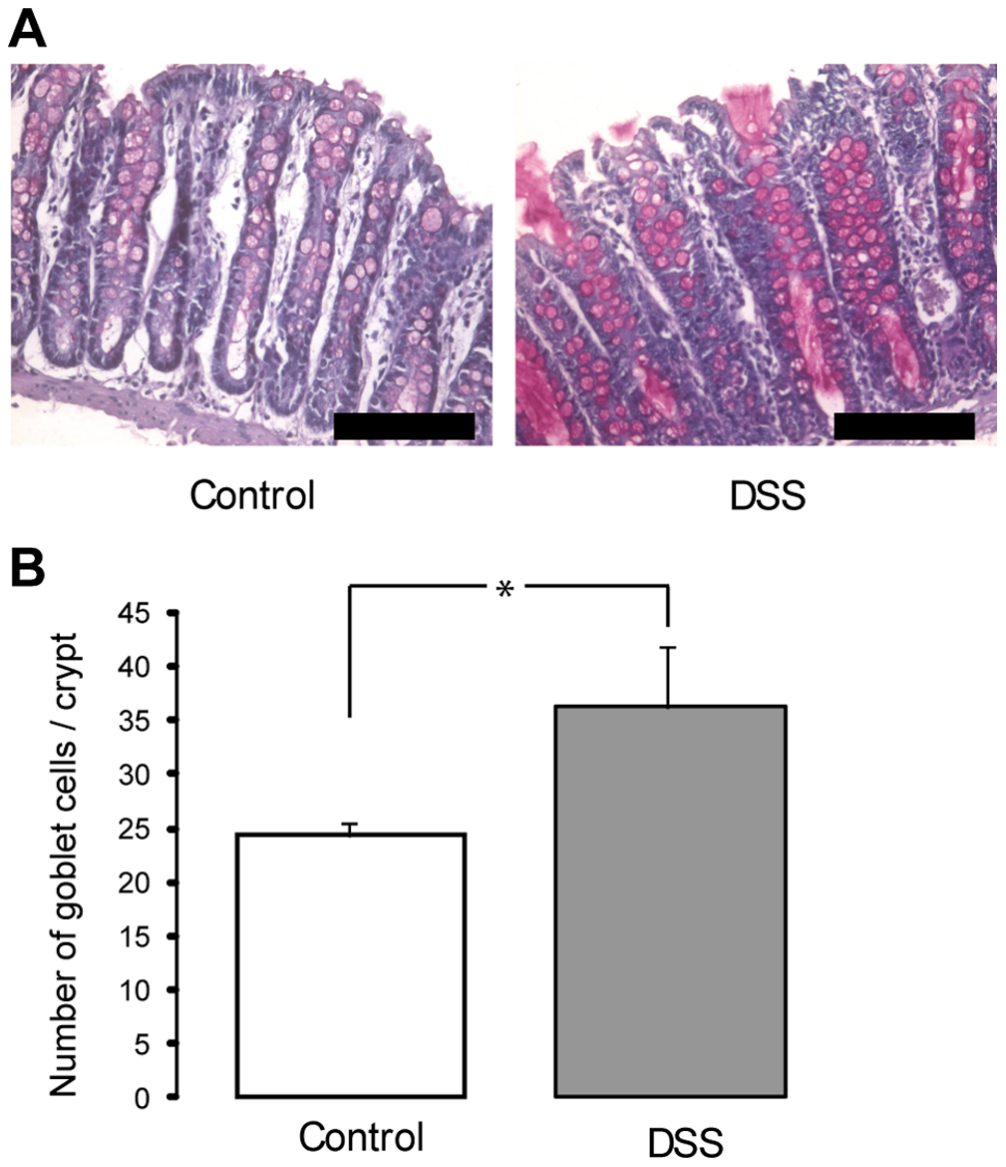
Goblet cell count in control- and DSS-treated rats. (**A**) PAS-staining of the colon of control rats (left) and DSS treated rats 8 days after colitis induction (right). Pink cells are PAS-positive. Bar 200 µm. (**B**) Quantification of the number of goblet cells per crypt (p<0.05). n = 24 in each group.

To investigate if the increased mucus thickness observed in colitic rats was a result of altered goblet cell numbers, PAS-staining of histological slides was performed (Fig. 2A). The PAS-staining demonstrated an increased number of PAS-positive cells per crypt, 8 days after DSS-induction of colitis, compared to untreated rats (Fig. 2A and B). There was no difference in average goblet cell size between untreated and colitic rats (270±69 vs 280±40 µm^2^, respectively). The thickness of the mucosa did not either differ between untreated and colitic rats (437±20 vs 420±70 µm, respectively).

### Inhibition of iNOS Decreased Mucus Thickness more in DSS Colitis than in Controls

**Figure 3:**
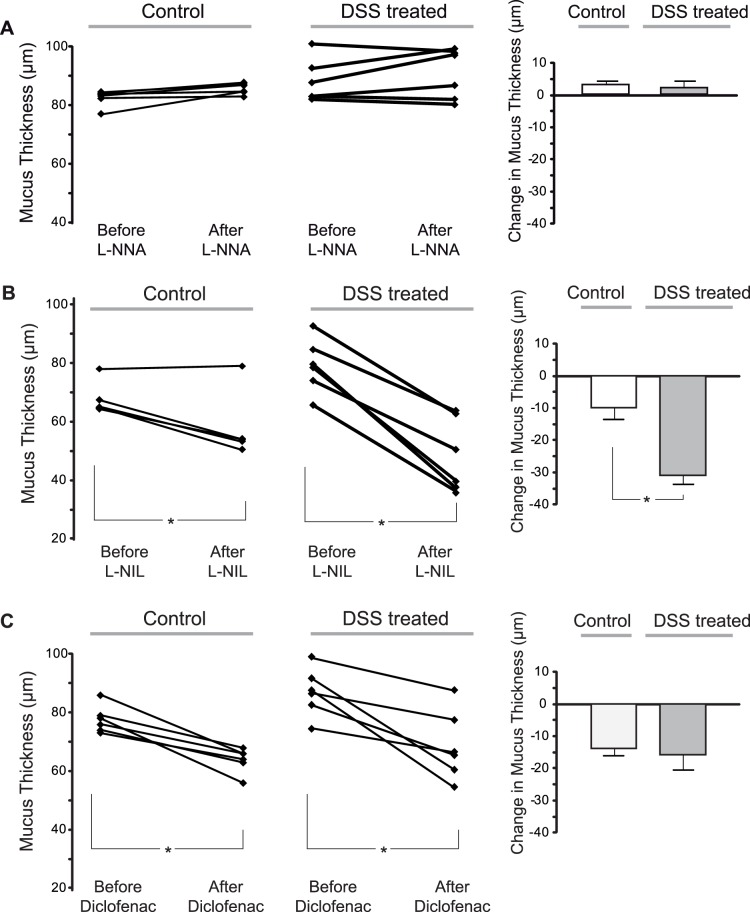
Firmly adherent mucus thickness in L-NNA-, L-NIL- and diclofenac-treated rats. Firmly adherent mucus thickness and mean changes in thickness in controls and DSS-colitic rats 60 minutes after the administration of either (**A**) L-NNA, (**B**) L-NIL or (**C**) diclofenac. (**A**) L-NNA had no effect on mucus thickness in either control or colitic rats**.** (**C**) Diclofenac decreased mucus thickness comparably in both groups while L-NIL reduced mucus thickness more in DSS-colitic than in control rats (**B**). (p<0.05). n = 6 in all groups.

The mechanisms leading to mucus secretion was then investigated by pharmacological inhibition of NO-synthases and the prostaglandin-generating enzyme cyclooxygenase, COX. The non-selective NOS-inhibitor L-NNA did not alter mucus thickness in either control (delta value +3±1 µm) or DSS-treated rats (delta value +3±2 µm, Fig. 3A). However, selective inhibition of the inflammatory NOS, iNOS, by L-NIL resulted in a significant decrease in basal mucus thickness (delta value −10±3 µm, Fig. 3B). This mucus decrease was even more pronounced in DSS-colitic rats (delta value −33±3 µm) and significantly greater compared to what was observed in control rats (Fig. 3A). Diclofenac-inhibition of COX resulted in a similar reduction of the firmly adherent mucus layer in control rats (delta value 14±2 µm) and in DSS-colitic rats (delta value −16±5 µm) (Fig. 3C). Further, inhibition of iNOS and COX reduced the mucus thickness similarly during basal (non-colitic) conditions. However, in DSS colitic rats, the mucus thickness was significantly more reduced by L-NIL than by diclofenac. The thickness of the loosely adherent mucus layers did not differ between groups. Before pharmacological intervention, the mean arterial blood pressure did not differ between the groups (range 85–95 mmHg) but L-NNA induced an increase in arterial blood pressure in both control (from 90±1 to 128±3 mmHg) and DSS-treated rats (from 87±2 to 129±3 mmHg), confirming an effect on eNOS by L-NNA but not L-NIL.

### iNOS and Prostaglandins Regulate Mucus Thickness in Distal Colon of Mice

**Figure 4:**
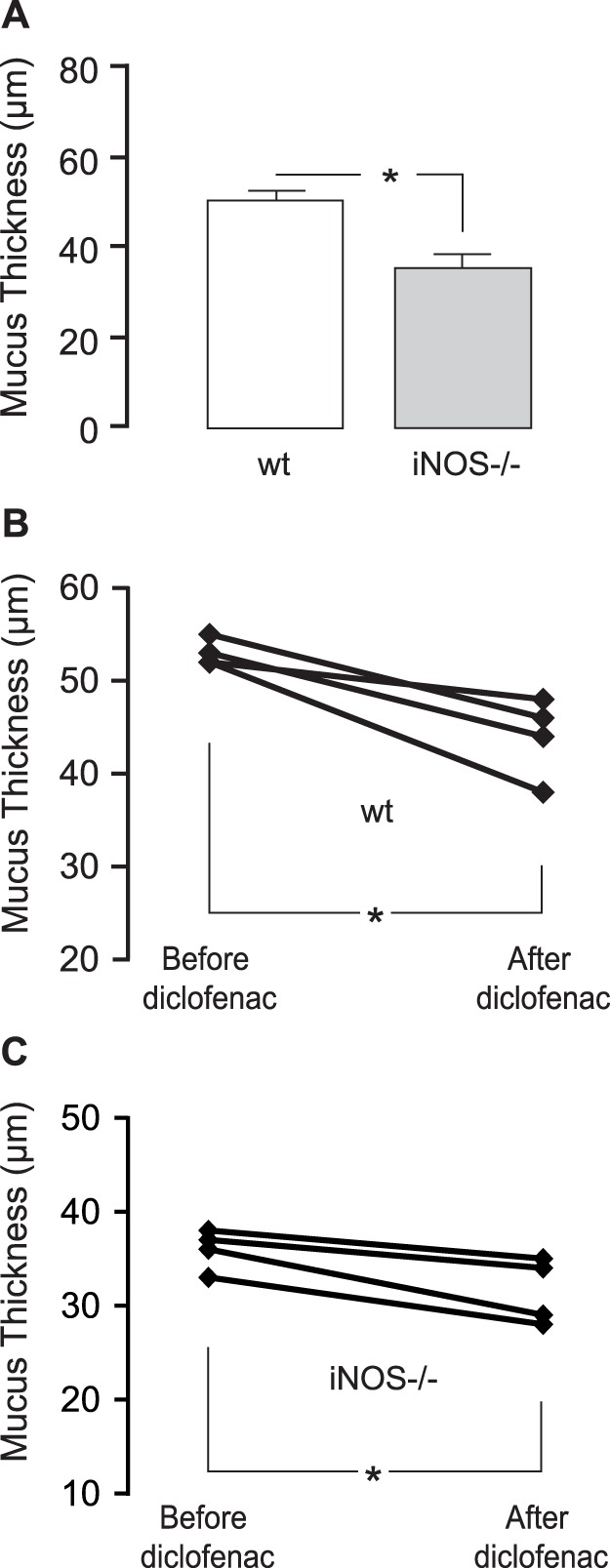
Firmly adherent mucus thickness in iNOS −/− and diclofenac-treated mice. (**A**) Firmly adherent mucus thickness in wild-type (wt) C57Bl/6 (n = 14) and iNOS −/− (C57Bl/6x129SvEv) mice (n = 9). (**B**) Wild-type C57Bl/6 and (**C**) iNOS −/− (C57Bl/6x129SvEv) mice before and 60 minutes after administration of diclofenac (n = 4 in each group).

To expand the observation of a regulatory role of iNOS-generated NO and prostaglandins in mucus secretion, the thickness of the colonic firmly adherent mucus layer was measured in iNOS deficient and diclofenac-treated mice. As previously reported, wild-type mice have a thinner firmly adherent colonic mucus layer (50±2 µm, Fig. 4) than rats (76±1 µm). Mice deficient in iNOS had a significantly thinner mucus layer (35±3 µm) than wild-type mice (Fig. 4A), confirming the results from the rat after L-NIL-inhibition of iNOS (Fig. 3B). The role of prostaglandins in regulating basal colonic mucus secretion was also similar in rats and mice, since COX-inhibition reduced the mucus thickness with −9±4 µm (Fig. 4B). In addition, COX inhibition in iNOS−/− mice further reduced mucus thickness slightly but significantly (−5±2 µm) indicating both a serial and parallel effect by nitric oxide and prostaglandins (Fig. 4C).

### Induction of Colitis Increased the Levels of iNOS in the Colonic Epithelium

**Figure 5:**
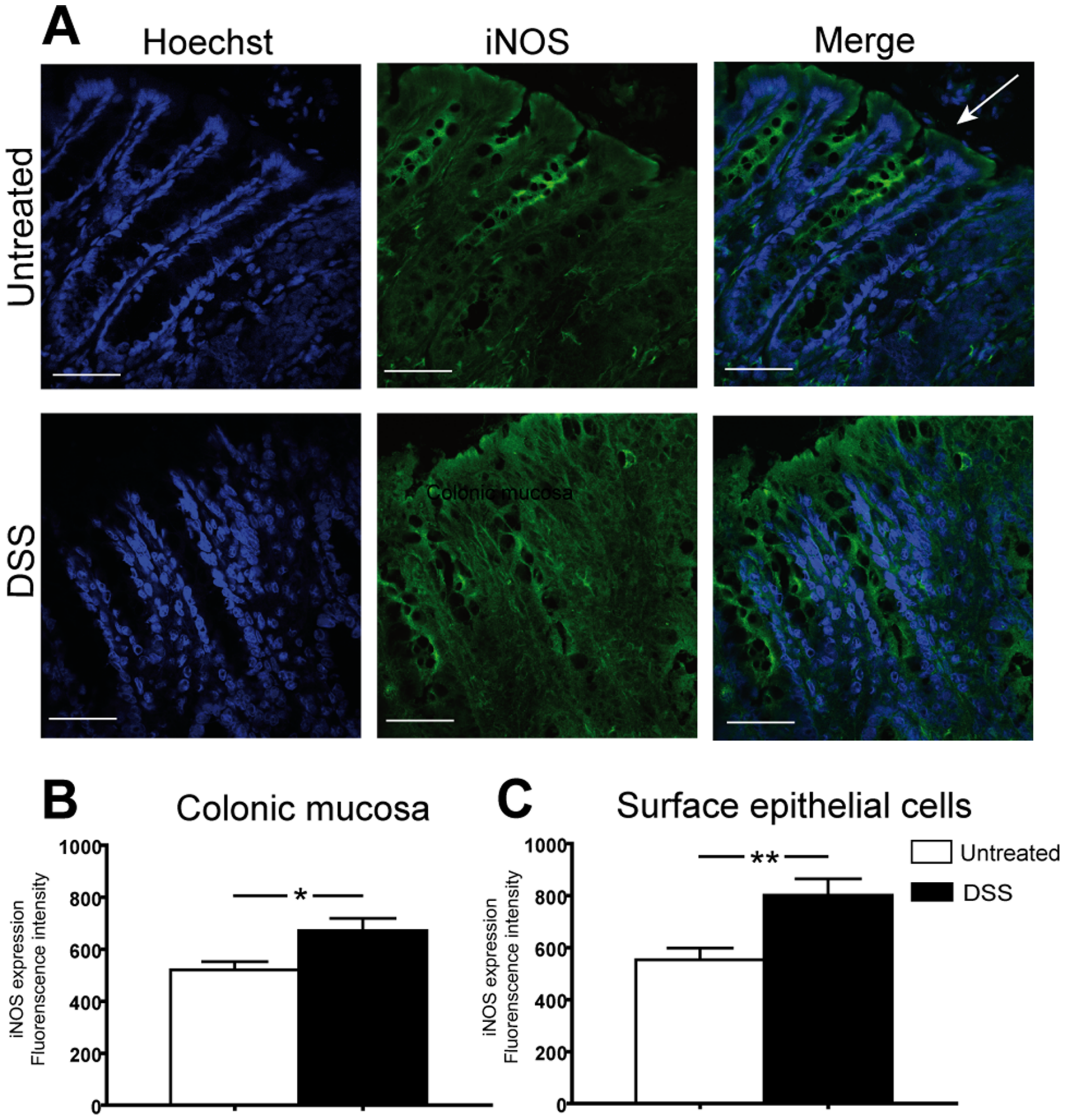
iNOS protein expression in colon after DSS treatment. (**A**) Cryosections of distal colon from untreated or DSS-treated animals were stained with iNOS-specific antibodies fluorescently detected by Alexa 488 secondary antibody. Bar corresponds to 50 µm and arrow marks luminal epithelium. Mean values of fluorescence intensity of iNOS protein in colonic mucosa (**B**) and surface epithelial cells (**C**) was measured and expressed as mean ± SE (n = 4). Statistical analysis was performed using an unpaired t-test with Welch’s correction.

Immunohistochemical evaluation of sectioned distal colon from untreated rats demonstrated that iNOS was constitutively expressed in the crypt cells and weakly expressed in the surface epithelium. During established DSS-induced colitis, the iNOS level was substantially increased in the surface epithelium and slightly in the crypt area (Fig. 5).

## Discussion

The present study demonstrates that the thickness of the firmly adherent colonic mucus was reduced during onset of DSS-induced colitis, which was followed by a significantly increased mucus thickness during established colitis in rats. This *in vivo* observation was supported by histological data showing increased number of PAS-positive mucus-producing goblet cells in the inflamed colonic mucosa. We found that iNOS was responsible for the increased mucus thickness observed in colitic animals, but was also involved in baseline mucus secretion, while the COX-system contributed equally to regulating mucus thickness in health and disease. Further, immunohistochemical evaluation revealed low constitutive levels of iNOS in the colonic mucosa and epithelium, which was substantially up regulated, mainly in the epithelium, during colitis.

The colonic mucus layer constitutes a continuous barrier that limits bacterial contact with the underlying epithelium. The importance of this barrier in intestinal homeostasis is evident from studies in mice deficient in the gel-forming colonic mucin Muc2, who spontaneously develop colitis and colorectal tumors [4], [5]. Furthermore, elimination of core 3-derived O-glycans in mice (C3GnT−/−) induce a reduction in Muc2 protein, resulting in a greater susceptability to DSS induced colitis as well as to colorectal adenocarcinoma [21]. Altered O-glycosylation of MUC2 mucins have also been shown to occur in sigmoid biopsies from patients with active ulcerative colitis [22].

In rectal biopsies from patients with flaring ulcerative colitis, goblet cell depletion and decreased colonic mucus thickness is observed [23], which contradict the typical disease symptoms of frequent blood- and mucus-containing loose stools [15], [16], [17]. This contradiction can be explained by the heterogeneity of the colonic inflammation resulting in goblet cell depletion in inflamed areas while increased goblet cell area and mucus secretion was detected in other parts of the colon, as suggested by Nakano et al [24]. Indeed, other studies show that biopsies from un-inflamed areas from ulcerative colitis patients has unaltered mucus thickness compared to observations made in control persons, while the inflamed sites has significantly thinner mucus [16], [17], perhaps as a result of local reduction or total loss of goblet cells. In addition, Strugala et al. [15] found that reduced goblet cell density and mucus thickness was only evident in persons with severe ulcerative colitis.

The present study investigates the fate of the colonic mucus layer during onset of DSS-induced colitis in rats, and a reduction of the firmly adherent mucus layer was observed in the distal colon already one day following addition of DSS to the drinking water. However, established DSS-colitis in mice has previously been demonstrated to correlate with reduced mucus thickness [3]. The DSS-treated mice exhibited a greater disease activity [3] compared to the rats in this study, indicating that the concomitant mucosal destruction during severe colitis prohibits a compensatory increase of the mucus barrier. If the reduced mucus thickness observed in rats the first two days following DSS administration is due to deterioration of the mucus gel properties is not known, but the reduced thickness could allow luminal bacteria or other inflammatory agents to penetrate the mucus and induce colitis. In mice, a direct effect of DSS on the mucus biophysical structure has been observed after 12 h of DSS treatment, resulting in a loss of the stratified lamellar appearance of the firmly adherent mucus and a massive bacterial contact with the epithelium [25].

Following the initial reduction in colonic mucus thickness and a potential immune activation by penetrating bacteria, mucus thickness increases in colitic rats 3–8 days after DSS administration in parallel with increased goblet cell number and mucus secretion. This strengthening of the mucus barrier is likely to be a protective mechanism to reduce epithelial contact with luminal agents. Indeed, NFκB (Nuclear factor kappa B), a key component in colonic inflammation [26], can directly increase mucus secretion through binding to the Muc2 promotor region [27]. Other investigators have reported goblet cell depletion [24] in inflamed areas in the distal colon, and loss of goblet cells in the vicinity of lesions [28] in DSS induced colitis in rats. When goblet cell depletion was found in inflamed areas, a compensatory increase in goblet cell size and mucus secretion was measured in the proximal part of colon [24]. Furthermore, loss of goblet cells in the vicinity of lesions [28] was accompanied by an increase in Muc2 mRNA and fractional synthesis rate of mucins. The contradictory results of apparent mucus cell depletion combined with increased mucus production and mucus cell turnover in two models of experimental colitis in guinea pigs [29] was explained by the premature discharge of mucin making the mucus cells unrecognizable by light microscopy. These results explain why an increase in mucus production in colitis may occur even in the presence of mucus cell depletion. Further, Renes et al [30] found reduced number of goblet cells during active DSS-induced colitis accompanied by maintained Muc2 precursor synthesis and total level of secreted Muc2 in the distal colon. These results indicate an increased Muc2 synthesis per goblet cell. In addition, Muc2 secretion was observed to increase when the rats were in a regenerative phase. Thus, these observations suggest a maintained or even elevated barrier function during DSS colitis, which supports our data.

iNOS is a hallmark of inflammation [31], but the present study demonstrates low constitutive levels of iNOS protein also during basal conditions in the colonic epithelium, which has previously been reported at the levels of mRNA for the colonic [32], duodenal [33] and gastric mucosa [34]. DSS-induced colitis up regulated the levels of iNOS protein in the surface epithelial cells, which is consistent with our previously published data reporting increased mRNA levels of iNOS and eNOS, but not nNOS in the colon of DSS-treated rats [11]. The iNOS up regulation per se during inflammation is not surprising since iNOS has been localized in infiltrating neutrophils and macrophages in the colonic mucosa and submucosa in animal models of IBD [35] as well as in IBD patients [36], [37]. However, the localization of iNOS within the colonic epithelial cells also during basal, non-inflammatory conditions indicates a regulatory function of this enzyme during homeostasis. Indeed, a role of iNOS in maintaining baseline colonic mucus secretion was demonstrated in the present study both using pharmacological specific inhibition in rats, but also in iNOS deficient mice. During established DSS colitis, the role of iNOS was even more extensive, since iNOS was responsible for the increased colonic mucus thickness seen during DSS colitis. eNOS and nNOS on the other hand, did not seem to be involved in the regulation of mucus secretion, as L-NNA, a potent inhibitor of eNOS and nNOS but a much less efficient inhibitor of iNOS [38], in a dose that increase mean arterial blood pressure and potently reduced colonic blood flow [11], did not influence colonic mucus secretion in rats. This is in contrast to a report by Vallance et al. [39], which suggested that eNOS is an important regulator of mucus secretion based on the finding of fewer and less mucus filled goblet cells in eNOS-deficient mice. However, when we measured mucus thickness *in vivo* in eNOS-deficient mice in a pilot study, mucus thickness was similar to that observed in wild-type mice (44±4 µm, n = 2 versus 50±2 µm, respectively), but thicker than in iNOS-deficient mice (35±3 µm).

Prostaglandins have been shown to be involved in stimulating colonic mucus secretion [12], [40]. Here we show that prostaglandins are important in maintaining baseline colonic mucus secretion but not in stimulating mucus secretion following DSS treatment. Okayama et al [13] showed that endogenous prostaglandins afford protection against colonic ulceration in DSS induced colitis, which might be explained partly by their ability to maintain the mucus barrier.

In conclusion, DSS-induced mild colitis in rats initially disrupts the mucus barrier, which is followed by an increase in mucus secretion, probably stimulated by epithelial iNOS and consequently a strengthening of the mucus barrier. While prostaglandins regulated mucus secretion similarly in untreated and colitic rats, iNOS derived nitric oxide was shown to be important not only in baseline mucus secretion, but also in the mucus thickness increase observed in colitic rats. Our study provides new insights into the mechanisms of DSS-induced colitis as well as providing insight into basic physiology and pathophysiology of colonic mucus secretion.

## References

[1] Allen A, Hutton DA, Pearson JP (1998) The MUC2 gene product: a human intestinal mucin. The international journal of biochemistry & cell biology 30: 797–801. 9722984 10.1016/s1357-2725(98)00028-4

[2] Johansson MEV, Phillipson M, Petersson J, Velcich A, Holm L, et al (2008) The inner of the two Muc2 mucin-dependent mucus layers in colon is devoid of bacteria. Proceedings of the National Academy of Sciences of the United States of America 105: 15064–15069. 18806221 10.1073/pnas.0803124105 PMC2567493

[3] Petersson J, Schreiber O, Hansson GC, Gendler SJ, Velcich A, et al (2011) Importance and regulation of the colonic mucus barrier in a mouse model of colitis. American journal of physiology Gastrointestinal and liver physiology 300: G327–333. 21109593 10.1152/ajpgi.00422.2010 PMC3302190

[4] Velcich A, Yang W, Heyer J, Fragale A, Nicholas C, et al (2002) Colorectal cancer in mice genetically deficient in the mucin Muc2. Science (New York, NY) 295: 1726–1729. 10.1126/science.1069094 11872843

[5] Van der Sluis M, De Koning BAE, De Bruijn ACJM, Velcich A, Meijerink JPP, et al (2006) Muc2-deficient mice spontaneously develop colitis, indicating that MUC2 is critical for colonic protection. Gastroenterology 131: 117–129. 16831596 10.1053/j.gastro.2006.04.020

[6] Atuma C, Strugala V, Allen A, Holm L (2001) The adherent gastrointestinal mucus gel layer: thickness and physical state in vivo. American journal of physiology Gastrointestinal and liver physiology 280: G922–929. 11292601 10.1152/ajpgi.2001.280.5.G922

[7] Corfield AP, Wagner SA, Clamp JR, Kriaris MS, Hoskins LC (1992) Mucin degradation in the human colon: production of sialidase, sialate O-acetylesterase, N-acetylneuraminate lyase, arylesterase, and glycosulfatase activities by strains of fecal bacteria. Infection and immunity 60: 3971–3978. 1398908 10.1128/iai.60.10.3971-3978.1992 PMC257425

[8] Murthy SN, Cooper HS, Shim H, Shah RS, Ibrahim SA, et al (1993) Treatment of dextran sulfate sodium-induced murine colitis by intracolonic cyclosporin. Digestive diseases and sciences 38: 1722–1734. 8359087 10.1007/BF01303184

[9] Sartor RB (2008) Microbial influences in inflammatory bowel diseases. Gastroenterology 134: 577–594. 18242222 10.1053/j.gastro.2007.11.059

[10] Dicksved J, Schreiber O, Willing B, Petersson J, Rang S, et al.. (2012) Lactobacillus reuteri maintains a functional mucosal barrier during DSS treatment despite mucus layer dysfunction. PloS one 7. doi:10.1371/journal.pone.0046399. 10.1371/journal.pone.0046399 PMC3459901 23029509

[11] Petersson J, Schreiber O, Steege A, Patzak A, Hellsten A, et al (2007) eNOS involved in colitis-induced mucosal blood flow increase. American journal of physiology Gastrointestinal and liver physiology 293: G1281–1287. 17947450 10.1152/ajpgi.00357.2007

[12] Plaisancié P, Barcelo A, Moro F, Claustre J, Chayvialle JA, et al (1998) Effects of neurotransmitters, gut hormones, and inflammatory mediators on mucus discharge in rat colon. The American journal of physiology 275: G1073–1084. 9815038 10.1152/ajpgi.1998.275.5.G1073

[13] Okayama M, Hayashi S, Aoi Y, Nishio H, Kato S, et al (2007) Aggravation by selective COX-1 and COX-2 inhibitors of dextran sulfate sodium (DSS)-induced colon lesions in rats. Digestive diseases and sciences 52: 2095–2103. 17429720 10.1007/s10620-006-9597-z

[14] Phillipson M, Johansson MEV, Henriksnäs J, Petersson J, Gendler SJ, et al (2008) The gastric mucus layers: constituents and regulation of accumulation. American journal of physiology Gastrointestinal and liver physiology 295: G806–812. 18719000 10.1152/ajpgi.90252.2008

[15] Strugala V, Dettmar PW, Pearson JP (2008) Thickness and continuity of the adherent colonic mucus barrier in active and quiescent ulcerative colitis and Crohn’s disease. International journal of clinical practice 62: 762–769. 18194279 10.1111/j.1742-1241.2007.01665.x

[16] Pullan RD, Thomas GA, Rhodes M, Newcombe RG, Williams GT, et al (1994) Thickness of adherent mucus gel on colonic mucosa in humans and its relevance to colitis. Gut 35: 353–359. 8150346 10.1136/gut.35.3.353 PMC1374589

[17] Fyderek K, Strus M, Kowalska-Duplaga K, Gosiewski T, Wedrychowicz A, et al (2009) Mucosal bacterial microflora and mucus layer thickness in adolescents with inflammatory bowel disease. World journal of gastroenterology: WJG 15: 5287–5294. 19908336 10.3748/wjg.15.5287 PMC2776855

[18] Swidsinski A, Loening-Baucke V, Theissig F, Engelhardt H, Bengmark S, et al (2007) Comparative study of the intestinal mucus barrier in normal and inflamed colon. Gut 56: 343–350. 16908512 10.1136/gut.2006.098160 PMC1856798

[19] Cooper HS, Murthy SN, Shah RS, Sedergran DJ (1993) Clinicopathologic study of dextran sulfate sodium experimental murine colitis. Laboratory investigation; a journal of technical methods and pathology 69: 238–249. 8350599

[20] Holm L, Phillipson M (2012) Assessment of mucus thickness and production in situ. Methods in molecular biology (Clifton, NJ) 842: 217–227. 10.1007/978-1-61779-513-8_12 22259138

[21] An G, Wei B, Xia B, McDaniel JM, Ju T, et al (2007) Increased susceptibility to colitis and colorectal tumors in mice lacking core 3-derived O-glycans. The Journal of experimental medicine 204: 1417–1429. 17517967 10.1084/jem.20061929 PMC2118614

[22] Larsson JMH, Karlsson H, Crespo JG, Johansson MEV, Eklund L, et al (2011) Altered O-glycosylation profile of MUC2 mucin occurs in active ulcerative colitis and is associated with increased inflammation. Inflammatory bowel diseases 17: 2299–2307. 21290483 10.1002/ibd.21625

[23] Jacobs LR, Huber PW (1985) Regional distribution and alterations of lectin binding to colorectal mucin in mucosal biopsies from controls and subjects with inflammatory bowel diseases. The Journal of clinical investigation 75: 112–118. 3965499 10.1172/JCI111662 PMC423415

[24] Nakano S, Ohara S, Kubota T, Saigenji K, Hotta K (1999) Compensatory response of colon tissue to dextran sulfate sodium-induced colitis. Journal of gastroenterology 34: 207–214. 10213120 10.1007/s005350050245

[25] Johansson MEV, Gustafsson JK, Sjöberg KE, Petersson J, Holm L, et al.. (2010) Bacteria penetrate the inner mucus layer before inflammation in the dextran sulfate colitis model. PloS one 5. doi:10.1371/journal.pone.0012238. 10.1371/journal.pone.0012238 PMC2923597 20805871

[26] Murano M, Maemura K, Hirata I, Toshina K, Nishikawa T, et al (2000) Therapeutic effect of intracolonically administered nuclear factor kappa B (p65) antisense oligonucleotide on mouse dextran sulphate sodium (DSS)-induced colitis. Clinical and experimental immunology 120: 51–58. 10759763 10.1046/j.1365-2249.2000.01183.x PMC1905625

[27] Lee H-W, Ahn D-H, Crawley SC, Li J-D, Gum JR Jr, et al (2002) Phorbol 12-myristate 13-acetate up-regulates the transcription of MUC2 intestinal mucin via Ras, ERK, and NF-kappa B. The Journal of biological chemistry. 277: 32624–32631. 10.1074/jbc.M200353200 12077118

[28] Faure M, Moënnoz D, Montigon F, Mettraux C, Mercier S, et al (2003) Mucin production and composition is altered in dextran sulfate sodium-induced colitis in rats. Digestive diseases and sciences 48: 1366–1373. 12870797 10.1023/a:1024175629909

[29] Kaftan SM, Wright NA (1989) Studies on the mechanisms of mucous cell depletion in experimental colitis. The Journal of pathology 159: 75–85. 2809887 10.1002/path.1711590115

[30] Renes IB, Boshuizen JA, Van Nispen DJPM, Bulsing NP, Büller HA, et al (2002) Alterations in Muc2 biosynthesis and secretion during dextran sulfate sodium-induced colitis. American journal of physiology Gastrointestinal and liver physiology 282: G382–389. 11804861 10.1152/ajpgi.00229.2001

[31] Kubes P, McCafferty DM (2000) Nitric oxide and intestinal inflammation. The American journal of medicine 109: 150–158. 10967157 10.1016/s0002-9343(00)00480-0

[32] Perner A, Andresen L, Normark M, Rask-Madsen J (2002) Constitutive expression of inducible nitric oxide synthase in the normal human colonic epithelium. Scandinavian journal of gastroenterology 37: 944–948. 12229970 10.1080/003655202760230919

[33] Holm M, Powell T, Casselbrant A, Johansson B, Fändriks L (2001) Dynamic involvement of the inducible type of nitric oxide synthase in acid-induced duodenal mucosal alkaline secretion in the rat. Digestive diseases and sciences 46: 1765–1771. 11508680 10.1023/a:1010674109111

[34] Phillipson M, Henriksnäs J, Holstad M, Sandler S, Holm L (2003) Inducible nitric oxide synthase is involved in acid-induced gastric hyperemia in rats and mice. American journal of physiology Gastrointestinal and liver physiology 285: G154–162. 12646421 10.1152/ajpgi.00432.2002

[35] Miller MJ, Thompson JH, Zhang XJ, Sadowska-Krowicka H, Kakkis JL, et al (1995) Role of inducible nitric oxide synthase expression and peroxynitrite formation in guinea pig ileitis. Gastroenterology 109: 1475–1483. 7557128 10.1016/0016-5085(95)90633-9

[36] Palatka K, Serfozo Z, Veréb Z, Hargitay Z, Lontay B, et al (2005) Changes in the expression and distribution of the inducible and endothelial nitric oxide synthase in mucosal biopsy specimens of inflammatory bowel disease. Scandinavian journal of gastroenterology 40: 670–680. 16036527 10.1080/00365520510015539

[37] Zhang XJ, Thompson JH, Mannick EE, Correa P, Miller MJ (1998) Localization of inducible nitric oxide synthase mRNA in inflamed gastrointestinal mucosa by in situ reverse transcriptase-polymerase chain reaction. Nitric oxide: biology and chemistry/official journal of the Nitric Oxide Society 2: 187–192. 10.1006/niox.1998.0177 9731636

[38] Moncada S, Higgs A, Furchgott R (1997) International Union of Pharmacology Nomenclature in Nitric Oxide Research. Pharmacological reviews 49: 137–142. 9228663

[39] Vallance BA, Dijkstra G, Qiu B, van der Waaij LA, van Goor H, et al (2004) Relative contributions of NOS isoforms during experimental colitis: endothelial-derived NOS maintains mucosal integrity. American journal of physiology Gastrointestinal and liver physiology 287: G865–874. 15217783 10.1152/ajpgi.00187.2004

[40] Wallace JL (2001) Prostaglandin biology in inflammatory bowel disease. Gastroenterology clinics of North America 30: 971–980. 11764538 10.1016/s0889-8553(05)70223-5

